# Supplementary material for: A Likelihood Approach for Real-Time Calibration of Stochastic Compartmental Epidemic Models
Source: PLoS Comput Biol. 2017 Jan 17;13(1):e1005257. doi: 10.1371/journal.pcbi.1005257 (PMC5240920; doi:10.1371/journal.pcbi.1005257)
Supplement: S1 File — (TAR.GZ) [file pcbi.1005257.s014.tar.gz › HSPH_Online-SI-Revision/Additions/EnKF08/n100-extreme-SEITR_noisy/n100-extreme-SEITR_noisy_EnKF2_table-4.pdf]

1	newly 3 weeks spec	13577.1	538.52	{13., 705.}	96.0336	1.79481	newly 3 weeks cumul	31325.2	13774.7	{1613., 13616.}	105.288	0.915499	newly 5 weeks spec	12047.4	75.09	{0., 182.}	99.3767	2.8973	newly 5 weeks cumul	57070.8	14037.3	{1627., 13899.}	97.1312	1.12205
																			newly 54 weeks cumul	86564.8	14102.2	{1627., 13924.}	93.9467	1.27523
2	newly 3 weeks spec	17235.	1629.97	{256., 4802.}	90.5427	1.35239	newly 3 weeks cumul	29177.	22813.	{8937., 69857.}	73.2571	0.414588	newly 5 weeks spec	17453.	310.	{4., 1192.}	98.2238	2.5668	newly 5 weeks cumul	67910.	23813.9	{9070., 70247.}	70.7808	0.651776
																			newly 62 weeks cumul	97717.	24080.8	{9079., 70255.}	76.9923	0.792251
3	newly 3 weeks spec	14424.	7252.2	{910., 20473.}	66.5336	0.582091	newly 3 weeks cumul	24222.	43671.7	{10958., 93400.}	110.79	0.328748	newly 5 weeks spec	18266.	2134.79	{83., 4462.}	88.3128	1.35438	newly 5 weeks cumul	61841.	49611.3	{12223., 108670.}	59.6855	0.376879
																			newly 64 weeks cumul	97783.	53260.7	{12284., 119965.}	55.5432	0.450336
4	newly 3 weeks spec	13822.	968.77	{308., 1785.}	92.9911	1.30225	newly 3 weeks cumul	25848.	23772.	{9421., 67669.}	73.102	0.365543	newly 5 weeks spec	16022.	76.3	{4., 190.}	99.5238	2.69535	newly 5 weeks cumul	59339.	24097.7	{9589., 67988.}	65.6232	0.568224
																			newly 62 weeks cumul	94062.	24145.1	{9599., 67995.}	74.4266	0.742943
5	newly 3 weeks spec	22578.	7034.16	{986., 15148.}	69.6936	0.6839	newly 3 weeks cumul	36477.	49127.4	{12479., 121557.}	81.5738	0.298423	newly 5 weeks spec	16920.	1648.5	{79., 3587.}	90.2571	1.32623	newly 5 weeks cumul	77798.	54049.7	{14202., 124990.}	58.0581	0.388608
																			newly 64 weeks cumul	97923.	56357.6	{14915., 125156.}	54.8275	0.417139
6	newly 3 weeks spec	20143.	2695.43	{0., 8412.}	86.6185	2.77648	newly 3 weeks cumul	34123.	38135.9	{7165., 71049.}	59.321	0.253516	newly 5 weeks spec	17405.	1002.92	{0., 3733.}	94.2377	3.43686	newly 5 weeks cumul	74866.	40711.2	{7397., 79596.}	58.2637	0.4269
																			newly 63 weeks cumul	93450.	43021.3	{7436., 108138.}	61.1732	0.483708
7	newly 3 weeks spec	12108.	559.07	{0., 1047.}	95.8534	3.24332	newly 3 weeks cumul	28975.	11149.3	{1939., 12374.}	83.6637	0.722057	newly 5 weeks spec	11332.	150.83	{0., 539.}	98.669	3.57145	newly 5 weeks cumul	52923.	11565.7	{2101., 12695.}	85.3061	0.919315
																			newly 55 weeks cumul	88800.	12118.7	{2167., 12695.}	87.1491	1.08978
8	newly 3 weeks spec	16980.	1346.75	{323., 2150.}	92.0686	1.33273	newly 3 weeks cumul	32632.	23302.4	{8562., 59193.}	69.3908	0.430438	newly 5 weeks spec	14766.	171.33	{7., 247.}	98.8397	2.50842	newly 5 weeks cumul	65812.	23936.6	{8695., 59328.}	67.7841	0.617705
																			newly 62 weeks cumul	97109.	24049.1	{8705., 59328.}	76.2369	0.774086
9	newly 3 weeks spec	23304.	5671.19	{739., 10835.}	76.3807	0.885686	newly 3 weeks cumul	35304.	41118.4	{8493., 102289.}	76.0903	0.335729	newly 5 weeks spec	17411.	1619.02	{57., 3739.}	90.7012	1.53004	newly 5 weeks cumul	78660.	45651.4	{8592., 122299.}	60.7729	0.464946
																			newly 64 weeks cumul	98833.	48240.5	{8596., 122329.}	59.7858	0.500923
10	newly 3 weeks spec	12030.	2341.41	{1., 3865.}	95.159	2.0666	newly 3 weeks cumul	25011.	9042.9	{1010., 26349.}	90.3418	1.02131	newly 5 weeks spec	13614.	816.6	{0., 1035.}	94.0018	2.86377	newly 5 weeks cumul	52825.	11420.8	{1010., 29170.}	88.7085	1.26475
																			newly 60 weeks cumul	95503.	12335.	{1010., 30113.}	87.9355	1.46766
11	newly 3 weeks spec	13896.	1703.22	{161., 7231.}	87.9515	1.50223	newly 3 weeks cumul	26326.	9108.56	{1037., 22489.}	94.7192	0.995944	newly 5 weeks spec	14675.	380.04	{14., 1849.}	97.4103	2.18104	newly 5 weeks cumul	57151.	10281.1	{1277., 29837.}	88.1147	1.22811
																			newly 59 weeks cumul	97210.	10854.9	{1454., 31101.}	88.9387	1.41318
12	newly 3 weeks spec	16108.	2390.91	{1., 3282.}	94.0877	2.16145	newly 3 weeks cumul	33166.	7551.35	{722., 20505.}	86.1301	1.14121	newly 5 weeks spec	14159.	1014.52	{0., 307.}	93.3351	2.93705	newly 5 weeks cumul	64453.	10336.9	{722., 28337.}	87.0222	1.36169
																			newly 60 weeks cumul	95054.	11508.5	{722., 28361.}	87.8927	1.50046
13	newly 3 weeks spec	15592.	686.29	{0., 958.}	95.5984	2.33737	newly 3 weeks cumul	30732.	7610.74	{620., 18192.}	79.0907	0.875367	newly 5 weeks spec	14704.	175.93	{0., 223.}	98.8035	3.13458	newly 5 weeks cumul	62511.	8127.04	{694., 19167.}	87.0847	1.14748
																			newly 61 weeks cumul	96828.	8320.45	{714., 19167.}	91.407	1.32904
14	newly 3 weeks spec	10081.	868.	{1., 930.}	95.4284	2.17829	newly 3 weeks cumul	23444.	11082.	{972., 17557.}	100.798	0.943469	newly 5 weeks spec	11642.	203.99	{0., 186.}	98.2478	3.35748	newly 5 weeks cumul	46832.	11714.	{972., 18527.}	92.0067	1.17676
																			newly 54 weeks cumul	89533.	11877.3	{972., 18715.}	90.1454	1.4194
15	newly 3 weeks spec	14236.	725.45	{0., 1169.}	95.4339	3.19884	newly 3 weeks cumul	30841.	11758.3	{1392., 13386.}	79.5336	0.74236	newly 5 weeks spec	13230.	307.48	{0., 557.}	97.6759	3.48283	newly 5 weeks cumul	59486.	12513.1	{1520., 13386.}	83.3167	0.954279
																			newly 55 weeks cumul	91749.	13507.8	{1559., 13386.}	86.5854	1.08769
16	newly 3 weeks spec	11254.	855.69	{13., 1040.}	92.3966	1.70031	newly 3 weeks cumul	24092.	6004.56	{81., 8686.}	84.0854	1.10441	newly 5 weeks spec	12953.	265.13	{0., 649.}	97.9531	2.52881	newly 5 weeks cumul	50447.	6735.25	{96., 8722.}	87.0088	1.3344
																			newly 58 weeks cumul	87287.	7268.2	{104., 8726.}	91.6732	1.52487
17	newly 3 weeks spec	15615.	6264.5	{881., 12159.}	66.2913	0.643506	newly 3 weeks cumul	24517.	46131.4	{9890., 117832.}	125.608	0.358169	newly 5 weeks spec	20574.	1815.19	{72., 4615.}	91.1773	1.50325	newly 5 weeks cumul	68260.	51129.2	{11339., 121121.}	61.8405	0.419113
																			newly 64 weeks cumul	92364.	54377.7	{11805., 121327.}	56.8372	0.452541
18	newly 3 weeks spec	19007.	2222.4	{0., 8109.}	88.3075	2.39098	newly 3 weeks cumul	31170.	34357.4	{7002., 69593.}	63.24	0.299996	newly 5 weeks spec	17189.	904.57	{0., 3354.}	94.7375	3.23221	newly 5 weeks cumul	70235.	36572.7	{7284., 69593.}	59.0957	0.463514
																			newly 63 weeks cumul	98793.	39753.5	{7320., 69602.}	62.9334	0.544857
19	newly 3 weeks spec	12889.	893.3	{21., 612.}	93.4142	1.67042	newly 3 weeks cumul	24953.	4908.12	{90., 5747.}	86.6266	1.07558	newly 5 weeks spec	14542.	319.04	{3., 318.}	97.8061	2.42844	newly 5 weeks cumul	54787.	5753.39	{137., 5903.}	90.0319	1.33333
																			newly 58 weeks cumul	96606.	6510.16	{185., 5930.}	93.2611	1.53393
20	newly 3 weeks spec	19368.	2223.09	{0., 8378.}	88.5218	2.43757	newly 3 weeks cumul	33454.	39522.7	{7179., 89544.}	71.3139	0.342132	newly 5 weeks spec	16864.	564.31	{0., 2740.}	96.6538	3.28633	newly 5 weeks cumul	72041.	41090.6	{7394., 106307.}	57.8631	0.472015
																			newly 63 weeks cumul	97397.	42566.2	{7436., 115519.}	61.5494	0.553002
21	newly 3 weeks spec	19672.	6382.13	{814., 17516.}	69.2499	0.774362	newly 3 weeks cumul	30439.	43210.9	{3456., 96648.}	95.5822	0.410333	newly 5 weeks spec	18543.	1767.57	{160., 3580.}	90.4677	1.40479	newly 5 weeks cumul	73114.	48200.4	{4662., 106934.}	58.6315	0.485237
																			newly 64 weeks cumul	98246.	51232.8	{6200., 112758.}	54.8215	0.516576
22	newly 3 weeks spec	14910.	2041.66	{0., 1137.}	95.5426	2.22508	newly 3 weeks cumul	31688.	6973.88	{950., 23314.}	88.7382	1.16982	newly 5 weeks spec	14003.	901.73	{0., 225.}	93.9444	2.96559	newly 5 weeks cumul	61831.	9450.69	{950., 42178.}	87.8658	1.38894
																			newly 60 weeks cumul	92206.	10433.2	{950., 42178.}	88.6849	1.53471
23	newly 3 weeks spec	16317.	489.45	{0., 508.}	97.0004	2.35949	newly 3 weeks cumul	34910.	6834.	{1182., 19441.}	81.6759	0.932735	newly 5 weeks spec	13260.	156.2	{0., 81.}	98.822	3.23305	newly 5 weeks cumul	65072.	7257.26	{1313., 19458.}	88.8473	1.17093
																			newly 61 weeks cumul	83725.	8078.19	{1342., 19521.}	90.3515	1.25741
24	newly 3 weeks spec	16757.	2776.2	{9., 8395.}	84.7193	1.72258	newly 3 weeks cumul	25641.	37942.	{7206., 88906.}	92.041	0.338194	newly 5 weeks spec	20012.	866.43	{0., 3401.}	95.6704	3.18806	newly 5 weeks cumul	69280.	40263.8	{7440., 105148.}	59.393	0.46894
																			newly 63 weeks cumul	98228.	42446.2	{7486., 114001.}	62.0575	0.555454
25	newly 3 weeks spec	12372.	1892.88	{59., 10650.}	88.5618	1.57222	newly 3 weeks cumul	23919.	9749.68	{326., 17823.}	103.517	1.14036	newly 5 weeks spec	15457.	516.91	{11., 1507.}	96.7179	2.27368	newly 5 weeks cumul	55191.	11331.1	{379., 62455.}	90.4566	1.36785
																			newly 59 weeks cumul	91517.	12134.7	{413., 69677.}	88.1016	1.50673
26	newly 3 weeks spec	12283.	2633.	{229., 11499.}	87.0607	1.25271	newly 3 weeks cumul	28429.	13203.1	{1220., 38501.}	102.837	0.989546	newly 5 weeks spec	11730.	1064.15	{63., 1377.}	91.7416	1.63881	newly 5 weeks cumul	53475.	15873.6	{1622., 55195.}	92.0324	1.12477
																			newly 57 weeks cumul	80102.	18829.7	{1943., 85826.}	86.9003	1.18102
27	newly 3 weeks spec	12409.	2429.83	{241., 13088.}	85.2645	1.28095	newly 3 weeks cumul	28757.	10759.7	{1111., 30063.}	96.9956	1.04602	newly 5 weeks spec	11908.	1294.29	{100., 1452.}	91.2663	1.62004	newly 5 weeks cumul	53686.	13842.2	{1403., 51270.}	89.0444	1.16754
																			newly 57 weeks cumul	90968.	17422.1	{1633., 84419.}	84.4165	1.24237
28	newly 3 weeks spec	16959.	6753.76	{999., 20072.}	66.5444	0.591805	newly 3 weeks cumul	28083.	42096.	{11785., 92756.}	88.3634	0.29311	newly 5 weeks spec	18063.	2083.51	{127., 3591.}	88.4653	1.26778	newly 5 weeks cumul	68202.	47825.9	{12552., 105025.}	57.2117	0.37398
																			newly 64 weeks cumul	88415.	51439.9	{12894., 108880.}	54.8702	0.39812
29	newly 3 weeks spec	12914.	507.8	{0., 478.}	98.075	2.56888	newly 3 weeks cumul	24569.	7766.92	{931., 18944.}	79.8821	0.835029	newly 5 weeks spec	15388.	185.27	{0., 82.}	98.796	3.48287	newly 5 weeks cumul	56007.	8250.95	{1003., 18964.}	88.6951	1.15951
																			newly 61 weeks cumul	96532.	8614.66	{1009., 18964.}	91.9067	1.3802
30	newly 3 weeks spec	13125.	6009.38	{1216., 10991.}	63.2029	0.51979	newly 3 weeks cumul	20142.	45299.9	{9572., 121710.}	148.906	0.349169	newly 5 weeks spec	19690.	1599.41	{156., 3436.}	91.877	1.40224	newly 5 weeks cumul	60177.	49847.8	{14170., 125454.}	61.4466	0.360409
																			newly 64 weeks cumul	98710.	52604.6	{14262., 125725.}	56.0462	0.434469
31	newly 3 weeks spec	10370.	768.3	{16., 1165.}	93.0748	1.68158	newly 3 weeks cumul	26082.	15457.5	{1585., 54439.}	106.599	0.82014	newly 5 weeks spec	9931.	119.35	{0., 245.}	98.7982	2.84011	newly 5 weeks cumul	46868.	15867.2	{1590., 59613.}	93.5518	0.993605
																			newly 54 weeks cumul	76589.	15983.3	{1590., 60195.}	87.8166	1.15376
32	newly 3 weeks spec	12614.	2627.9	{0., 5967.}	96.2188	2.29445	newly 3 weeks cumul	26998.	8057.2	{863., 34183.}	87.017	1.06709	newly 5 weeks spec	13001.	1046.32	{0., 4917.}	91.952	2.98083	newly 5 weeks cumul	54036.	11046.9	{863., 35146.}	87.1223	1.29504
																			newly 60 weeks cumul	93822.	12331.8	{863., 66750.}	86.8562	1.48782
33	newly 3 weeks spec	20264.	6060.19	{752., 15148.}	70.493	0.798032	newly 3 weeks cumul	37971.	40087.	{9731., 97417.}	71.7305	0.364383	newly 5 weeks spec	15166.	1883.75	{47., 3740.}	87.5791	1.41965	newly 5 weeks cumul	73450.	45236.6	{10585., 108839.}	59.9198	0.464733
																			newly 64 weeks cumul	96763.	48434.3	{10618., 113013.}	58.045	0.507981
34	newly 3 weeks spec	15602.	2656.8	{0., 10274.}	93.7307	2.52985	newly 3 weeks cumul	34185.	6812.65	{1124., 21250.}	86.5342	1.20292	newly 5 weeks spec	13645.	1058.88	{0., 6177.}	92.2398	3.11138	newly 5 weeks cumul	63916.	9878.18	{1124., 41781.}	87.3644	1.40999
																			newly 60 weeks cumul	89965.	10810.8	{1124., 52492.}	87.9834	1.53022
35	newly 3 weeks spec	18683.	6393.6	{985., 20689.}	69.1976	0.73943	newly 3 weeks cumul	29482.	44142.5	{9603., 95918.}	90.2767	0.350051	newly 5 weeks spec	19217.	1524.74	{91., 3671.}	92.0657	1.53721	newly 5 weeks cumul	73185.	48676.3	{10019., 111447.}	58.6269	0.457395
																			newly 64 weeks cumul	94833.	50839.5	{10031., 115994.}	55.7793	0.490997
36	newly 3 weeks spec	11929.	1587.21	{0., 909.}	98.3964	2.25052	newly 3 weeks cumul	21930.	5163.69	{1069., 4729.}	89.1536	1.06829	newly 5 weeks spec	15024.	612.86	{0., 270.}	95.9208	3.06832	newly 5 weeks cumul	52068.	6954.23	{1069., 10116.}	91.0662	1.37113
																			newly 60 weeks cumul	97587.	7959.03	{1069., 25472.}	91.8442	1.60183
37	newly 3 weeks spec	15965.	640.12	{0., 813.}	95.9905	2.40795	newly 3 weeks cumul	25084.	8079.22	{825., 20462.}	78.4105	0.839677	newly 5 weeks spec	18951.	160.78	{0., 154.}	99.1516	3.45596	newly 5 weeks cumul	65780.	8559.29	{922., 20477.}	87.2311	1.21011
																			newly 61 weeks cumul	98650.	8753.02	{945., 20477.}	91.1272	1.37754
38	newly 3 weeks spec	14686.	1005.02	{13., 4560.}	93.1566	1.94154	newly 3 weeks cumul	31691.	6291.93	{154., 22648.}	88.4379	1.41475	newly 5 weeks spec	13535.	328.72	{0., 1029.}	97.5713	2.58866	newly 5 weeks cumul	60924.	7182.61	{208., 26932.}	89.7399	1.60141
																			newly 58 weeks cumul	87770.	7717.4	{338., 28805.}	91.2072	1.69024
39	newly 3 weeks spec	14352.	1408.99	{0., 646.}	96.7235	2.75361	newly 3 weeks cumul	28679.	4327.15	{764., 4840.}	90.1835	1.22606	newly 5 weeks spec	15042.	648.18	{0., 257.}	95.6909	3.3277	newly 5 weeks cumul	60279.	6071.34	{764., 7395.}	91.9438	1.49286
																			newly 60 weeks cumul	93593.	7193.62	{770., 7803.}	92.3139	1.65209
40	newly 3 weeks spec	14498.	579.7	{0., 491.}	96.0015	2.6166	newly 3 weeks cumul	31327.	7049.07	{1004., 17480.}	81.3244	0.902948	newly 5 weeks spec	13194.	272.46	{0., 79.}	97.935	3.50605	newly 5 weeks cumul	59514.	7718.13	{1134., 17480.}	87.5257	1.15154
																			newly 61 weeks cumul	94658.	8249.91	{1173., 17480.}	91.2845	1.34338
41	newly 3 weeks spec	13435.	6998.08	{1032., 16223.}	63.2984	0.546847	newly 3 weeks cumul	19619.	50830.3	{11964., 114974.}	179.224	0.390595	newly 5 weeks spec	23349.	1929.27	{70., 4110.}	91.7372	1.52854	newly 5 weeks cumul	66595.	56302.1	{12687., 118317.}	61.697	0.37478
																			newly 64 weeks cumul	95882.	59390.7	{13351., 118935.}	53.7075	0.414111
42	newly 3 weeks spec	10315.	1326.37	{0., 1199.}	97.0803	3.12373	newly 3 weeks cumul	25161.	11754.5	{323., 12359.}	81.3748	0.763945	newly 5 weeks spec	10541.	599.81	{0., 625.}	96.117	3.45855	newly 5 weeks cumul	46907.	13200.5	{401., 13638.}	85.4818	0.96117
																			newly 55 weeks cumul	87719.	15165.6	{421., 16089.}	87.5157	1.15281
43	newly 3 weeks spec	12045.	2091.43	{0., 3974.}	93.9368	2.13164	newly 3 weeks cumul	24152.	7462.34	{1091., 25599.}	89.2868	1.03963	newly 5 weeks spec	14237.	898.9	{0., 1601.}	93.8535	2.90634	newly 5 weeks cumul	52694.	9927.55	{1112., 50879.}	86.8296	1.29159
																			newly 60 weeks cumul	95113.	10957.4	{1115., 54197.}	88.4796	1.50976
44	newly 3 weeks spec	16196.	1628.98	{0., 1392.}	94.757	2.40848	newly 3 weeks cumul	32797.	6624.31	{1127., 15857.}	88.6249	1.18706	newly 5 weeks spec	14170.	622.34	{0., 264.}	95.608	3.13537	newly 5 weeks cumul	64055.	8407.12	{1127., 16522.}	89.1732	1.41748
																			newly 60 weeks cumul	95957.	9292.32	{1127., 26195.}	90.3162	1.56546
45	newly 3 weeks spec	16795.	1030.54	{283., 1732.}	93.864	1.40032	newly 3 weeks cumul	31747.	21232.7	{8633., 48083.}	61.4401	0.401373	newly 5 weeks spec	14996.	126.35	{5., 212.}	99.1574	2.65882	newly 5 weeks cumul	65154.	21693.7	{8772., 48123.}	67.2143	0.611794
																			newly 62 weeks cumul	96985.	21786.2	{8772., 48123.}	77.5366	0.780287
46	newly 3 weeks spec	15111.	5942.87	{800., 14391.}	65.7332	0.601405	newly 3 weeks cumul	25292.	43043.2	{12393., 98074.}	102.911	0.319504	newly 5 weeks spec	17991.	1795.73	{50., 4126.}	90.0187	1.40585	newly 5 weeks cumul	63693.	47960.6	{14125., 111077.}	57.223	0.37413
																			newly 64 weeks cumul	97957.	51043.1	{14633., 114425.}	55.3171	0.448809
47	newly 3 weeks spec	13356.	1505.15	{12., 3081.}	91.1094	1.72284	newly 3 weeks cumul	27095.	7721.98	{73., 16275.}	94.5748	1.22276	newly 5 weeks spec	14547.	437.06	{2., 1218.}	96.9955	2.45523	newly 5 weeks cumul	57180.	8936.19	{82., 23683.}	90.5316	1.45348
																			newly 59 weeks cumul	92154.	10217.2	{86., 25973.}	90.3396	1.60697
48	newly 3 weeks spec	14508.	1011.27	{169., 1892.}	93.0296	1.37853	newly 3 weeks cumul	25267.	23553.8	{8873., 66498.}	72.477	0.385848	newly 5 weeks spec	17877.	135.61	{2., 213.}	99.2414	2.83297	newly 5 weeks cumul	62499.	24022.7	{8993., 66542.}	65.556	0.603909
																			newly 62 weeks cumul	95356.	24142.2	{8999., 66542.}	74.7432	0.767555
49	newly 3 weeks spec	15110.	1912.8	{0., 1329.}	96.1952	2.36439	newly 3 weeks cumul	34829.	5398.68	{904., 9670.}	88.7173	1.25564	newly 5 weeks spec	12499.	664.77	{0., 606.}	94.6814	2.85072	newly 5 weeks cumul	62570.	7407.8	{904., 9713.}	90.6075	1.45178
																			newly 60 weeks cumul	88106.	8211.04	{904., 14473.}	90.6805	1.55838
50	newly 3 weeks spec	13796.	294.32	{0., 523.}	97.8666	2.44664	newly 3 weeks cumul	28249.	9602.19	{860., 18663.}	85.968	0.875411	newly 5 weeks spec	14245.	60.38	{0., 81.}	99.5761	3.4297	newly 5 weeks cumul	58206.	9788.59	{948., 18677.}	87.5243	1.13859
																			newly 61 weeks cumul	94140.	9864.63	{985., 18677.}	89.8817	1.328
